# Near-Infrared Photoluminescent Carbon Nanotubes for Imaging of Brown Fat

**DOI:** 10.1038/srep44760

**Published:** 2017-03-20

**Authors:** Masako Yudasaka, Yohei Yomogida, Minfang Zhang, Takeshi Tanaka, Masako Nakahara, Norihiko Kobayashi, Yuko Okamatsu-Ogura, Ken Machida, Kazuhiko Ishihara, Kumiko Saeki, Hiromichi Kataura

**Affiliations:** 1 Nanomaterials Research Institute, National Institute of Advanced Industrial Science and Technology, Tsukuba, Ibaraki 305-8565, Japan; 2 CNT-Application Research Center, National Institute of Advanced Industrial Science and Technology, Tsukuba, Ibaraki 305-8565, Japan; 3 Department of Disease Control, Research Institute, National, Center for Global Health and Medicine, Shinjuku-ku, Tokyo 162-8655, Japan; 4 Department of Biomedical Sciences, Graduate School of Veterinary Medicine, Hokkaido University, Sapporo, 060-0818, Japan; 5 Department of Materials Engineering, The University of Tokyo, Hongo, Tokyo 113-8656, Japan

## Abstract

Near-infrared photoluminescent single-walled carbon nanotubes (CNTs) are expected to provide effectual bio-imaging tools, although, as yet, only limited applications have been reported. Here, we report that CNTs coated with an amphiphilic and biocompatible polymer, poly(2-methacryloyloxyethyl phosphorylcholine-*co*-*n*-butyl methacrylate; PMB), generate high-quality images of brown fat. Brown fat is a heat-productive adipose tissue, which is attracting increasing attention as a new therapeutic target for obesity-associated metabolic disorders. Its brown colour is mainly attributed to densely packed capillaries, which facilitate its high heat-exchanging efficiency. Currently, positron emission tomography-computed tomography is the only practical technique to identify brown fat distribution in the living body; however, it is expensive to use. By virtue of their high affinity to apolipoproteins and exemption from macrophage phagocytosis, PMB-CNTs selectively accumulate on capillary endothelial cells but not larger vessels in adipose tissue. Therefore, the image brightness of adipose tissue can directly reflect the capillary density, and indirectly the thermogenic capability and brownness. PMB-CNTs provide clearer images than conventional organic dyes, as the high level of transmitted light passes through the body with less light scattering. Thus, PMB-CNT-based imaging methods could open a new phase in thermogenic adipose tissue research.

Single-walled carbon nanotubes (CNTs)^1^,^2^, which emit near-infrared (NIR) fluorescence in the long wavelength region (1100–1400 nm: NIR-II)^3^, have been expected to provide excellent bio-imaging probes as they show deep penetration and low scattering in cells^4^,^5^ and the living body^6^,^7^,^8^,^9^,^10^,^11^,^12^. When intravenously injected, CNTs have successfully generated images of the hind limb vasculature of mice^10^, demonstrating the effectiveness of CNTs as an angiographic probe. Moreover, the 1300–1400 nm photoluminescence reportedly generates remarkably fine images of brain vasculature, demonstrating capillary tubes with high resolution, even at an immersion depth of 1 cm^11^. These recent advances illustrate the current trend towards the use of probes with longer wavelength photoluminescence (>1000 nm), which is becoming increasingly essential to obtain high-grade images in animal and clinical studies. CNTs have a safety advantage over the quantum dots used as NIR-II fluorescent materials, as quantum dots contain heavy metals^13^,^14^, while CNTs do not^1^,^2^. Nevertheless, technological advances are required to endow CNTs with the tissue targetability required for use in a broader spread of biomedical applications.

In this work, we used the amphiphilic and biocompatible polymer poly(2-methacryloyloxyethyl phosphorylcholine-*co*-*n*-butyl methacrylate) (PMB)^15^,^16^,^17^ to coat CNTs. The other worth property of PMB is that the PMB shows very small interactions with biological components such as proteins and cells, suggesting that the inflammatory immunological responses induced by PMB is quite low^17^. To observe the pharmacokinetics, we intravenously administered PMB-coated CNT (PMB-CNT) and found that the PMB-CNTs selectively accumulated in brown fats, providing clear NIR photoluminescent images of these tissues. The PMB-CNTs were exclusively located within the endothelial cells of capillaries, but not in the endothelial cells of other microvessels, including arterioles and venules. Brown fat exerts a high calorigenic effect and thus contributes to the maintenance of core body temperature under cold environments^18^.

Brown adipose tissue (BAT), a typical brown fat, is distributed in specific body areas including the interscapular region (iBAT), axillary region (aBAT), cervical region (cBAT), mediastinum (mBAT), and paravertebral region (prBAT)^19^,^20^. Brown adipocytes (BA) show unique subcellular structures; they are rich in mitochondria equipped with ladder-shaped dense cristae, reflecting their high capacity for oxidative phosphorylation. They also contain abundant multilocular lipid droplets (MLD), which are located in close vicinity to mitochondria, making the process of oxidative phosphorylation highly efficient^21^,^22^. Mitochondria possess uncoupled protein 1 (UCP1) on their inner membranes, a proton channel that promotes proton leakage from the intermembranous space to the mitochondrial matrix, deconstructing the proton gradient generated by the mitochondrial electron transport chain (mETC). After activation by beta-adrenergic receptor stimuli, UCP1 channels open and ATP production is hindered. Consequently, electrochemical potentials are released as heat energy^23^. A considerable number of reports have shown that BATs contribute to obesity prevention and metabolic improvement^21^,^24^,^25^,^26^,^27^,^28^, and can thus be considered as targets for the assessment and treatment of metabolic disorders.

Brown fat depots showing a lighter brown colour exist in the subcutaneous white adipose tissues (WAT) of the inguinal region (iWAT); these have been termed as brite^29^ or beige^30^,^31^ adipose tissues. Furthermore, certain portions of visceral WAT may also show a brown appearance, for example, upper portions of mesenteric WAT (mWAT) and lower portions of gonadal WAT (gWAT)^20^. Conversely, nearly the entire region of the retroperitoneal WAT (rpWAT) appears whitish, although it turns slightly brownish under a cold acclimated state^20^. Brite/beige adipocytes show morphologies intermediate between BA and white adipocytes (WA), with lesser numbers of uneven-sized lipid droplets^32^. Although individual brite/beige adipocytes show equivalent calorigenic capacities to BA^33^,^34^, UCP1-dependent oxygen consumption per gram of iWAT is maximally one-fifth of that of iBAT^31^. Consistent with these findings is the fact that capillary networks are not so densely developed in brite/beige depots as they are in iBATs, reflecting the light brownish colour of brite/beige depots. While invasive studies have revealed the locations of BAT, brite/beige, and WAT, and their different thermogenic functions, non-invasive observations on their differences are challenging.

Currently, positron emission tomography-computed tomography (PET-CT) using ^18^F-fluorodeoxy glucose (^18^F-FDG) is the only practical method for determining the distribution of brown fat in human and animal studies^35^. Since PET-CT examinations are expensive and require considerable work, the development of an optical imaging technique with a lower cost and higher feasibility is desirable. It is known that some organic probes are capable of brown fat imaging, these include indocyanine IR-786^36^, IRDye800 conjugated with a peptide selectively binding to vascular cells of BAT^37^, SRFluor680 enclosed in polyethylene glycol micelles^38^, and the curcumin analogue CRANAD-29^39^. However, such organic probes emit fluorescence at shorter wavelengths (700–900 nm), resulting in problems with low image resolution.

Since PMB-CNTs specifically accumulate in brown fat capillaries, and the density of the capillaries highly correlates with the heat-producing capacity of brown fat, PMB-CNTs may provide an ideal probe for brown fat imaging and estimation of the heat-production potential of such tissues. The molecular basis for PMB-CNT accumulation in brown fat endothelial cells is discussed with regard to the possibility of a complex formation between PMB-CNTs and apolipoproteins.

## Results

### Whole body NIR imaging of mice with PMB-CNT administration

**Figure 1:**
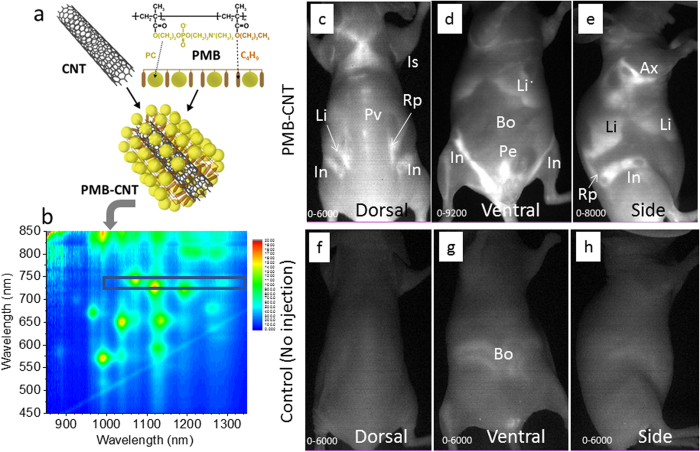
Schematics of CNT, PMB, and the supposed structure of PMB-coated CNT (**a**). PL spectra of PMB-CNT dispersed in water (**b**). The black rectangle in (**b**) denotes the excitation and PL wavelength region used in the imaging. Typical NIR-PL images of a mouse 5 h after i.v. injection of PMB-CNT (**c–e**) and those of a control mouse without injection (**f–h**). Is, interscapular; Pv, paravertebral; Ax, axillar; Rp, retroperitoneal; In, inguinal; Li, liver; Pe, pelvic; Bo, bowels. The PL intensity ranges are indicated in the bottom left of each image.

To gain NIR photoluminescence (PL) intensity of CNTs, fluorescent semiconducting-CNTs were separated from non-fluorescent metallic CNTs present in the HiPco specimens, via a previously described method^40^. The dispersant surfactants used for the separation were replaced to PMB via ultrafiltration^41^. The PMB-CNTs thus obtained (Fig. 1a) were dispersed almost individually in water, showing the characteristic sharp optical absorption peaks (Supplementary Fig. 1) and PL spectra (Fig. 1b) of CNTs. The PMB-CNT remained stably dispersed in water for several months (Supplementary Fig. 1), and the PL intensity of PMB-CNT did not decline after mixing with mouse blood serum or plasma (Supplementary Fig. 2). These findings suggest that PMB-CNTs are suitable for bio-imaging purposes.

PMB-CNT dispersed in water (CNT/water: 0.5 mg/mL) was intravenously (i.v.) administered to female nude mice via the tail vein at a dose of 0.15–0.2 mL/mouse. Typical NIR PL images of the whole mouse body taken at 5 h post-injection showed bright PL signals in the liver (Li) and interscapular (Is), axillar (Ax), paravertebral (Pv), inguinal (In), retroperitoneal (Rp), and pelvic (Pe) areas (n = 8; Fig. 1c–e; Supplementary Fig. 3). These sites coincided with the regions where adipose tissues are distributed, except for the liver, and similar results were also obtained from male mice (n = 3; Supplementary Fig. 4). By contrast, no PL signals were observed in control mice without PMB-CNT administration (n = 3) (Fig. 1f–h), except in bowel regions (Bo), where weak PL signals were attributed to intestinal contents (Fig. 1g).

### Histological confirmation of CNT accumulation in adipose tissues

**Figure 2:**
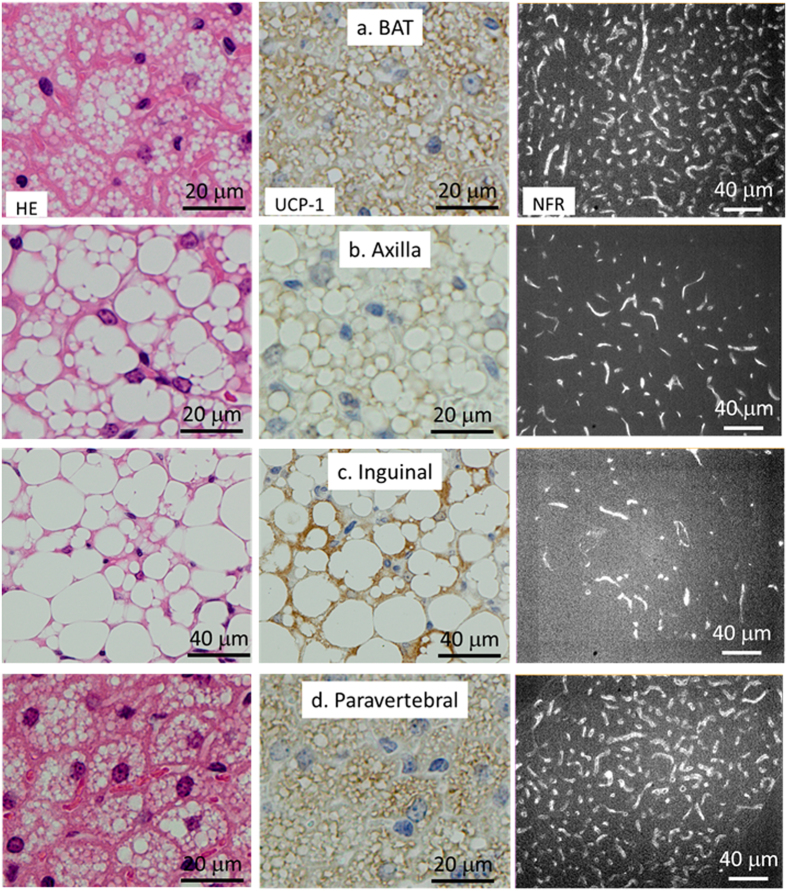
Histology of bright PL-emitting tissues. Adipose tissues from interscapular (**a**), axillar (**b**), inguinal (**c**), and paravertebral (**d**) areas of PMB-CNT-injected mice at a post-injection time of 3.5 h. Staining: HE (left), UCP-1 (centre), and NFR (right). Visible-light micrographs (left and centre) and NIR-PL micrographs (right).

Since particularly bright PL signals were detected in interscapular, axillar, and paravertebral regions, where BATs are located, and inguinal regions where brite/beige tissues are located, we hypothesised that CNTs preferentially accumulated in heat-producing adipose tissue. To verify this hypothesis, bright PL-emitting regions were analysed histologically. Cells from the interscapular, axillar, and paravertebral regions showed typical BA morphologies, with MLDs (Fig. 2a,b,d, left panels) and high-level UCP1 protein expression (Fig. 2a,b,d, centre panels), while lower numbers of UCP1^+^/MLD^+^ cells were observed in the inguinal region, consistent with the morphological characteristics of brite/beige depots (Fig. 2c, left and centre). To confirm the existence of CNTs in these adipose tissues, we observed tissue slices under NIR-PL microscopy, and confirmed the presence of PL signals with the intensities responding to the density of the capillary network of the adipose tissues (Fig. 2, right panels).

Similar results were obtained from tissues from the retroperitoneal and pelvic regions, which also showed strong PL (Supplementary Fig. 5). When dissected, parts of the mesenteric fat appeared bright under NIR-PL imaging, and tissue observations indicated that there were higher numbers of CNTs in the beige-like areas than in the WAT areas (Supplementary Fig. 5). Interscapular BAT tissue from control mice with no injection (n = 3) did not show any CNT-PL (Supplementary Fig. 6). Thus, CNTs accumulated primarily in BAT depots, to a lesser degree in brite/beige depots, and much less in pure WAT depots. Similar results were also obtained from male mice (n = 3; data not shown).

**Figure 3:**
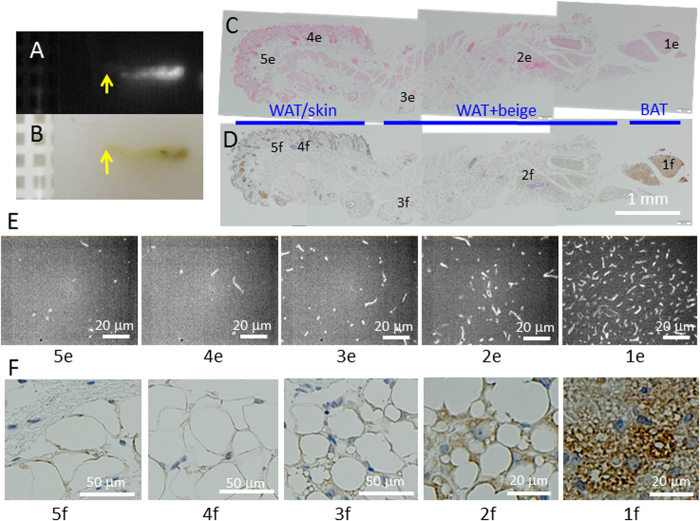
Detailed examinations of adipose tissues with CNT-accumulations. NIR-PL images (**A**) and naked-eye observations (**B**) of a paraffin block with a tissue cross-section from iBAT to skin. The tissue was collected from a PMB-CNT injected mouse at 3.5 h post-injection. Multi-field low magnification micrographs of the tissue slices stained with nuclear fast red (**C**) or an anti-UCP-1 antibody (**D**) are shown. High magnification NIR-PL micrographs (E, 1e-5e) and visible-light micrographs for UCP-1 staining (F, 1f-5f) are also presented.

To further confirm that the preferential accumulation of PMB-CNTs in adipose tissues occurred in association with the order of heat productivity (i.e., BAT >brite/beige >WAT), we examined PL signals from cross sections that covered interscapular to cutaneous regions. Whole tissue examinations revealed that the NIR-PL signal intensity (Fig. 3A) coincided with the brownness of the region, as observed with the naked eye (Fig. 3B). The lowest PL signals were associated with the lightest colours, which occurred in the superficial areas consisting of WAT (Fig. 3C,D, leftward), while medium PL signals were associated with a medium brownness in the sub-superficial areas that consisted of brite/beige adipose tissues (Fig. 3C,D, around the centre), and the highest PL signals were associated with the darkest brown occurring in the interscapular region that consisted of BAT (Fig. 3C,D, rightward). The high magnification micrographs are presented in Fig. 3E and F. Thus, CNT signals provided optical images of adipose tissues, reflecting their potential for heat-production.

### Accumulation of PMB-CNTs in capillary endothelial cells of adipose tissues

**Figure 4:**
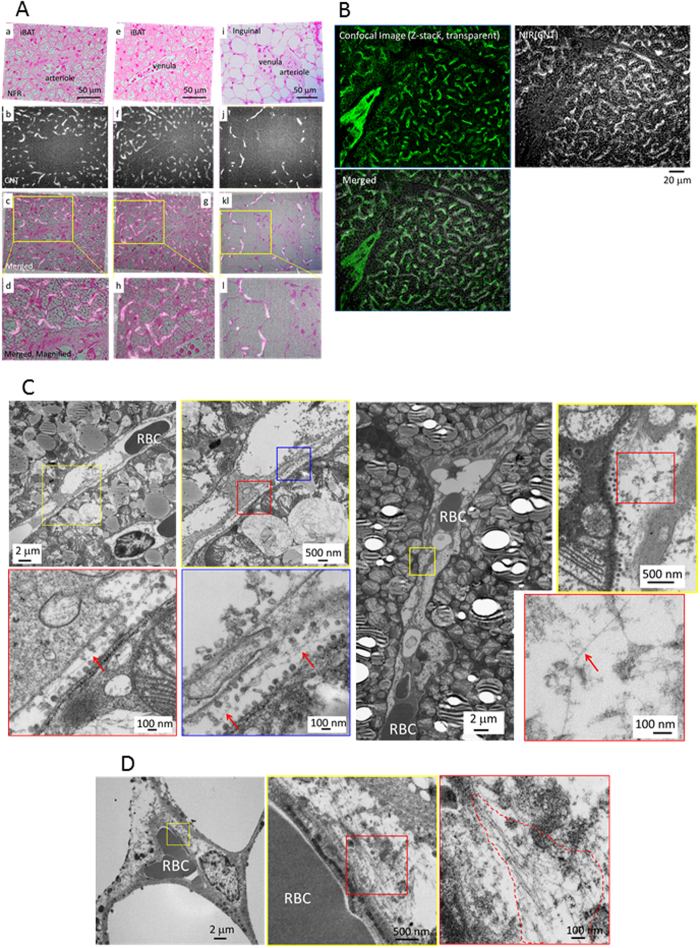
(**A**) Visible-light micrographs (a,e,i), NIR-PL micrographs (b,f,j), and merged images (c,g,k) with higher magnification (d,h,l), near an arteriole (a–d) and a venule (e–h) in iBAT, and near an arteriole/venule in iWAT (i–l). The tissues were obtained from mice at 3.5 h post-PMB-CNT injection (n = 3). Bright objects in NIR-PL images correspond to CNTs. Tissues were stained with NFR. (**B**) A confocal microscopy image, NIR-PL micrograph, and merged image of iBAT, obtained from a male mouse at 3 h post-PMB-CNT injection (n = 1). Endothelial cells were visualised by immunostaining with anti-CD31 antibody. **(C**,**D)** TEM observations to determine the localisation of CNTs in iBAT (**C**) and iWAT (**D**), obtained from a mouse at 3.5 h post-PMB-CNT injection (n = 1). Red arrows in (**C**) and an area enclosed by a red dotted line in (**D**) denote the existence of CNTs.

As the PL images showed capillary network-like structures (Fig. 2, right panels) demonstrating bright PL signals in inter-adipocyte spaces, but not in adipocytes *per se*, we hypothesised that PMB-CNTs accumulated in the capillaries of adipose tissues. This hypothesis was verified by detailed microscopic observations of iBAT, showing that PL signals coincided with the capillary structure (Fig. 4A). It was also noted that larger vessels, including arterioles and venules, were negative for PL signals, indicating that PMB-CNTs selectively accumulated in the capillaries of iBAT. Immunostaining studies using an anti-CD31 antibody confirmed that CNTs accumulated in the endothelial cells of the capillary network, but not in those of larger vessels (Fig. 4B). This PMB-CNT-based imaging profile was rather unexpected, as conventional CNTs effectively image blood vessels, and have been successfully used as angiographic probes^10^,^11^. This suggests that PMB modification endowed CNTs with a new property, which allows them to be used to selectively image capillary endothelial cells in adipose tissue.

To confirm the presence of CNTs in the capillary endothelial cells of adipose tissues, we also performed transmission electron microscopy (TEM) observations and determined that CNTs were present inside capillary endothelial cells in iBAT (Fig. 4C) and iWAT (Fig. 4D) at 3.5 h post-injection. In endothelial cells, CNTs were often lying individually, parallel to the capillary walls, or alternatively they were grouped into loose aggregations.

Thus, PMB-CNTs selectively accumulate in the endothelial cells of the capillary networks of adipose tissues, and the density of these capillaries closely correlates with the brown colour and heat exchangeability of heat-productive adipose tissues.

### Interaction between PMB-CNTs and apolipoproteins

On the basis of the findings on NIR-PL signals *in vivo* (Fig. 4), we hypothesised that PMB-CNTs have a specific affinity for capillary endothelial cells in adipose tissues. The capillary endothelial cells of adipose tissue reportedly have the unique ability to generate adipocytes *per se*, suggesting that adipocytes and capillary endothelial cells of adipose tissue share common characteristics^42^,^43^,^44^. Adipocytes are cells specialised in the uptake and storage of lipid compounds. Amphiphilic PMB has both polar phosphorylcholine groups and hydrophobic butyl groups in its side chains, and therefore endows a variety of synthetic materials with high biocompatibility on the basis of its ability to reduce protein adsorption and cell adhesion^45^. Therefore, PMB-CNTs may accumulate in capillary endothelial cells of adipose tissue by behaving like biogenic lipid compounds, such as serum lipids. CNTs (diameters = ca. 1 nm; lengths = 100–1000 nm) show comparable sizes to some serum lipoproteins, such as chylomicron remnants (CRs; diameter = 80 nm), very low density lipoproteins (VLDLs; diameter = 60 nm), and intermediate-density lipoproteins (IDLs; diameter = 40 nm). We therefore performed absorption assays to determine whether PMB-CNTs could interact with apolipoprotein B48 (ApoB48), apolipoprotein E (ApoE), and apolipoprotein C-II (ApoC-II). ApoB48 is required for chylomicron formation, ApoE mediates the interaction of CRs and VLDLs with their receptors, and ApoC-II is an activator of lipoprotein lipase.

**Figure 5:**
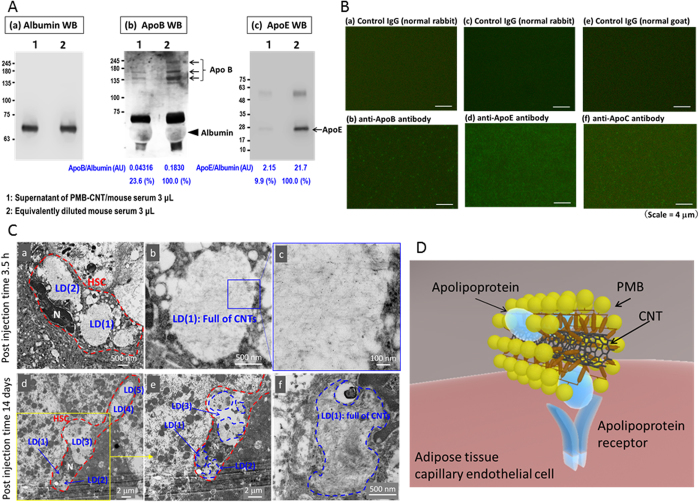
The specific interaction between PMB-CNTs and apolipoproteins. **(A**,**B)** Western blotting using the reaction of serum/PMB-CNTs (lane 1) and an equivalent volume of serum (lane 2) to detect albumin (a), ApoB (b), and ApoE (c). Albumin Western blotting was performed using 1/10 diluted samples. (**B**) Fluorescence images of PMB-CNT/serum mixture-smeared glass slides immunostained using control IgG (a, c: normal rabbit, e: normal goat), anti-ApoB antibody (b), anti-ApoE antibody (c), and anti-ApoC antibody (f). **(C)** TEM images of the liver of a PMB-CNT-administered mouse. Fine CNT structures were detected within lipid droplets (LDs) of stellate cells: LD(1) and LD(2) in (a–c), and LD(1), LD(2), and LD(3) in (d–f). CNTs were not observed in LD(4) and LD(5) in (d). HSC: hepatic stellate cell. Red and blue broken-lines denote HSC and the area where CNTs present, respectively. **(D)** A model for the binding of the CNT-PMB-apolipoprotein complex, with its receptor on the capillary endothelial cells of adipose tissue.

Murine serum was incubated with or without PMB-CNTs and the reaction supernatant was subjected to Western blotting. The amounts of ApoB48 and ApoE in the reaction supernatant were reduced by 76% and 90% respectively, via absorption by PMB-CNTs (Fig. 5A), thereby indicating that the larger parts of the ApoB48 and ApoE molecules on the CRs were transferred to PMB-CNTs. Positive findings were not obtained with ApoC-II because of the relatively low sensitivity of the assay (data not shown). To achieve a more sensitive assessment of the possible interaction between PMB-CNTs and ApoC, serum/PMB-CNT reaction mixtures were smeared onto glass slides and stained with anti-apolipoprotein antibodies. Although individual CNTs were invisible, numerous fine dots due to aggregations of PMB-CNTs were detected on the glass slides stained with anti-ApoB antibody (Fig. 5B,b) and anti-ApoE antibody (Fig. 5B,d). No such dots were detected on control IgG-stained glass slides (Fig. 5B,a,c) or serum-smeared slides (data not shown), verifying the specific interaction of PMB-CNTs with ApoB48 and ApoE. It was noticed that numerous fine dots were also detected on the glass slides stained with an anti-ApoC-II antibody (Fig. 5B,f), demonstrating that PMB-CNTs also interacted with ApoC-II. As ApoC-II is an activator of lipoprotein lipase, which is expressed in the capillary endothelial cells in tissues active in triglyceride metabolism, preferential accumulation of PMB-CNTs in the capillary endothelial cells of adipose tissue may be at least partly attributed to the ApoC/PMB-CNT interactivity.

It is also possible that ApoE/PMB-CNT interactivity may provide PMB-CNTs with access to the liver. To assess this possibility, TEM images of the livers of PMB-CNT-administered mice were examined in detail. At 3.5 h post-injection, CNT structures were detected in the sinusoid spaces (Supplementary Fig. 7A,B) and lipid droplets of hepatic stellate cells (HSCs; Fig. 5C,a–c, Supplementary Fig. 7C,D). These cells have a high capacity for uptake and storage of lipids and lipophilic compounds, including vitamin A. CNTs were more frequently observed in the lipid droplets of HSCs on Day 14 (Fig. 5C, d–f, Supplementary Fig. 8). By contrast, CNT structures were not detected in sinusoidal endothelial cells or Kupffer cells at either time point (data not shown). Concomitantly, NIR-PL micrographs of liver slices show broken net-shaped structures (Supplementary Fig. 9), which resemble neither the network structure of sinusoidal endothelial cells, nor the scattered distribution of macrophages. Thus, *in vivo* administered PMB-CNTs interacted with apolipoproteins in the blood stream, and by behaving like biogenic lipoproteins (Fig. 5D) they accumulated in specific body sites, depending on the characteristics of each apolipoprotein.

### NIR CNT-PL dynamics *in vivo*

**Figure 6:**
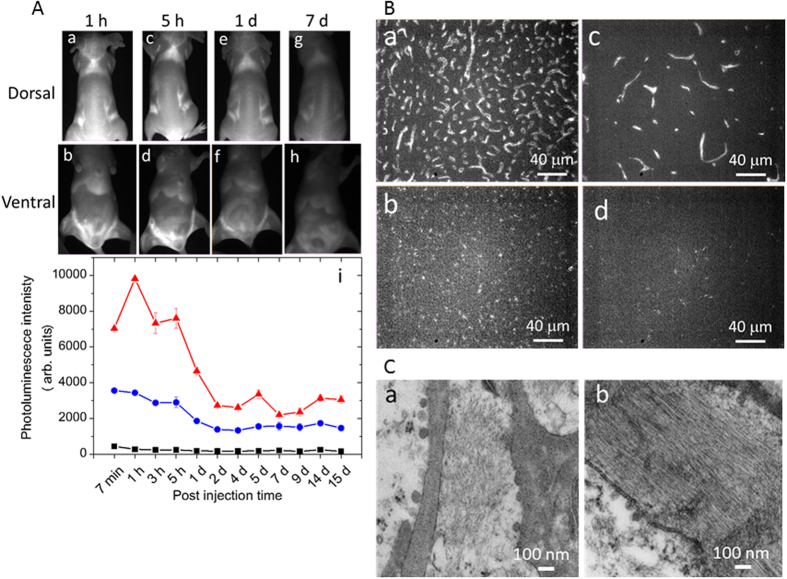
NIR CNT-PL dynamics *in vivo*. (**A**) Time course of whole body NIR PL images of a PMB-CNT-administered mouse (a–h), PL intensities in iBAT (red), a non-BAT area in the scapula (blue), and background (black). (i) PL intensities were measured at two to four places in each area. Error bars denote standard errors. Similar results were obtained from other mice (n = 5). (**B**) NIR PL micrographs of iBAT (a, b) and iWAT (Beige; c, d) at 3.5 hours (upper panels) and 14 days (bottom panels) post-injection (n = 3). (**C**) TEM images of iBAT (a) and iWAT (b) 14 days after injection (n = 1).

To determine the optical conditions for various applications of the PMB-CNT-based adipose tissue-imaging technique, we studied the *in vivo* dynamics of NIR CNT-PL intensity. Although bright PL signals were detected up to 24 h after PMB-CNT administration, the PL intensities in various adipose tissues declined thereafter (Fig. 6A,a–h). The PL intensities were quantitatively evaluated for non-BAT sites within the scapula and iBAT (Fig. 6A,i). After progressive decay of PL intensity up to Day 2 (T_1/2  _=1 day), low level PL signals were consistently emitted, at least up to Day 15, with these results being highly reproducible (Supplementary Fig. 10). The PL signal dynamics were further confirmed by micrographic examinations. After 3.5 h post-PMB-CNT administration, capillary endothelial cells in iBAT (Fig. 6B, panel a) and iWAT (Fig. 6B, panel c) were clearly observable with bright PL signals, whereas they had become indistinct by Day 14 (Fig. 6B, panels b and d). We found that, by Day 14, the CNTs had formed densely packed bundles within the capillary endothelial cells of adipose tissue (Fig. 6C, Supplementary Fig. 11), while they were less densely packed at 3.5 h post-injection (Fig. 4C,D). As CNT-CNT van der Waals interactions cause exciton annihilation^46^, and the excitation light and PL are absorbed by the densely-packed bundles of CNTs (self-shielding), PL signals from dense CNT-bundles are extremely weak. Similarly, PL signal intensities in the liver decreased over time (Fig. 6A, panels b,d,f,h). Thus, the optimal time point for adipose tissue imaging was identified as being within 1 day after PMB-CNT administration.

## Discussion

In this study, we have shown that PMB-CNTs are a unique NIR-PL imaging agent for the non-invasive visualisation of adipose tissues, and as PMB-CNTs accumulate in the endothelial cells, they directly reflect the capillary densities of such tissues, and indirectly reflect on the heat-production capacity of such tissues. In addition to whole body imaging, CNTs are of substantial utility for the detection of specific cell populations through microscopic observation of tissue slices.

Our success in the selective imaging of capillary endothelial cells of adipose tissue can be attributed to the PMB modification of CNTs. The structure of PMB strongly resembles the outer surface of plasma membranes and serum lipoproteins^45^. Therefore, in the blood stream, PMB-CNTs may behave like CRs, VLDL, or IDL. Nevertheless, PMB-CNTs are not genuine biogenic molecules, and thus cannot be incorporated into natural metabolic processes *in vivo*. As a result, they remain at the site to where they were guided by apolipoproteins. Consequently, ApoC-II-guided PMB-CNTs accumulated in capillary endothelial cells in adipose tissue, without being transported to adipocytes, and ApoE-guided PMB-CNTs accumulated at stellate LDs, without undergoing further transportation to hepatocytes.

Although the precise mechanisms remain elusive, the above findings do indicate that the adipose tissue capillary endothelial cells and liver stellate cells have distinct characteristics with regard to their affinity to lipoproteins. Additionally, sinusoidal endothelial cells are highly fenestrated, and therefore PMB-CNTs may easily escape from the blood stream to become trapped by stellate cells, which actively uptake lipoid compounds and store them in LDs.

In contrast to multi-walled carbon nanotubes, which have a similar size and shape to asbestos particles and are known to lead to serious health problems^47^,^48^, a consensus finding on the cytotoxicity of single-walled carbon nanotubes has yet to be obtained. Although we have not observed any obvious health problems in the PMB-CNT-administered mice, careful observations should be continued before PMB-CNT-based imaging techniques are applied in a broad range of animal studies focusing on advancing the understanding of adipose tissue biology.

## Methods

### Preparation of semiconducting CNT dispersion solutions

Around 100 mg of CNT(HiPco R1–831, NanoIntegris, Inc., Skokie, Il, USA) was mixed with 100 mL of an aqueous solution of sodium cholate (SC; 0.5% in weight; Sigma-Aldrich Co. LLC, St. Louis, MO, USA) and dispersed with a tip-type ultrasonic homogenizer (Sonifier 250D, Branson Ultrasonics, Emerson Japan, Ltd. Kanagawa, Japan) at 30 W/cm^2^ for 3 h. The dispersion solution was water-cooled during the ultrasonication. The obtained dispersion solution was centrifuged at 50 000 rpm (210 000 g) for 60 min in an angle rotor (S50A, Hitachi Koki Co., Ltd. Tokyo, Japan), and the supernatant (about 80 mL) was collected. Before separation, SDS (99%, Sigma-Aldrich Co. LLC) was added to the dispersion according to the separation condition. CNTs were separated from the supernatant using gel chromatography, as reported previously^40^. A chromatography system (AKTA explorer 10S, GE Healthcare, Chicago, IL, USA) was used for separation, with about 440 mL of allyl dextran-based gel beads (Sephacryl S-200 HR, GE Healthcare) packed in a column (Hiscale 50/200 column, GE Healthcare). About 50 mL of CNT dispersion with 0.5% SDS and 0.5% SC was applied to the column. After unbound metallic CNTs were eluted by applying 0.5% SDS and 0.5% SC solution, the adsorbed semiconducting CNTs were eluted and collected by applying 0.5% sodium deoxycholate (DOC) solution. The CNTs used for NIR imaging in this report were of a semiconducting type. About 15 mL of the obtained CNT solutions, with a concentration of 0.06 mg/mL (CNT/water), were used for the next process, with the DOC coating of the CNTs being replaced with SC, followed by PMB, which contained 30 mol% of the 2-methacryloyloxyethyl phosphorylcholine unit and 70 mol% of the BMA unit. The preparation method for PMB is described in refs 15 and 16. For the replacement with SC, ultrafiltration (Amicon Ultra 100 k, 15 mL, Merk Millipore Corp., Darmshtadt, Germany) was performed at 1000 g until the dispersion volume halved. The sediment was diluted with 0.5% SC solution and then centrifuged, with the process being repeated ten times. Next, the SC-CNT dispersion solution (15 mL) was mixed with 15 mg of PMB using a bath-type ultrasonic processor for 10 minutes, and then ultrafiltration (Amicon Ultra 3 k, 4 mL, Merck Millipore Corp.) was performed at 3000 g until the dispersion volume halved. The sediment was diluted with water and centrifuged, with the procedure repeated six times. The resultant PMB-CNT dispersion solution (approximately 1%PMB) was of 1.5–2 mL in quantity, and its CNT concentration was 0.5 mg/mL. The CNT concentration was estimated from the optical absorbance at 280 nm using a calibration line. The absorption spectra were measured with a UV-vis-NIR spectrometer (UV-3600, Shimadzu, Kyoto, Japan), and PL spectra were measured with a spectrofluorometer (NanoLog, HORIBA Ltd., Kyoto, Japan) equipped with a liquid nitrogen-cooled InGaAs array detector.

To find the influence of the blood components on the PL of CNT, the PMB-CNT was dispersed in mouse blood serum and plasma, to which the anticaking agents citric acid, heparin, and EDTA (Nippon Biotest Laboratories, Inc., Tokyo, Japan) were added. To mimic the mouse intravenous injection, 0.2 mL of the dispersion solutions (CNT: 0.5 mg/mL) was mixed with 2 mL of the blood serum or plasma, and PL spectra were measured 1 day after mixing.

### Near-infrared photoluminescence imaging

For the mouse imaging, a home-built imaging system^41^ was used. A lamp and NIR camera were placed obliquely above and just above the mouse, respectively. The lamp was LED (IP-307TCS, Lanics) used with an 800-nm short pass filter. The NIR camera (InGaAs-array video camera, NIRvana 640ST, Princeton Instruments, Trenton, NJ, USA) detected the CNT fluorescence through an objective lens (Cosmicar, Pentax, RICOH Imaging Company, Ltd., Tokyo, Japan) with the numerical aperture set to 2.8 was attached to the NIR camera window. A 1000 nm long-pass filter was placed in front of the objective lens to cut the excitation light and autofluorescence. The NIR camera exposure time was 100 ms. This imaging system was also used during the mouse dissection as a surgical guide to assist in finding the tissues in which CNT-PL was observed.

### Near-infrared fluorescence microscopy

NIR-PL observation was carried out using epi-illumination and optical microscopy (BX-51 IR, Olympus Corp., Tokyo, Japan). A Xe lamp (UXL-76XB, Ushio Inc., Tokyo Japan) was used to irradiate the specimen, with the light first passing through a single-band bandpass filter (708/75 nm; centre: 708 nm, width: 75 nm; BrightLine^®^, Semrock, IDEX Corp, Lake Forest, IL, USA) and being reflected by a 785 nm laser single-edge laser-flat dichroic beam splitter (BrightLine^®^, Semrock, IDEX Corp.). The PL emitted from the specimen passed through the 785 nm laser single-edge laser-flat dichroic beam splitter, an 808 nm best-value long-pass edge filter (EdgeBasic^TM^, Semrock, IDEX Corp), and a 1100 nm long-pass filter (Olympus), before reaching the NIR camera. The microscopy setup allowed visible-light transmission observation using the halogen lamp.

For NIR-PL microscopy observation, tissues were stained with nuclear fast red (NFR) because the NFR reagent does not emit NIR fluorescence with 708 nm excitation.

### Mouse experiments

Nude mice (BALB/cAJ1-nu/nu, Female; CLEA Japan Inc., Tokyo, Japan) were used in this study. They were allowed to acclimatise to the institute laboratory for 2–6 weeks before PMB-CNT was injected via tail veins. Doses of 0.15–0.2 mL/mouse (3.8–5 mg/kg CNT/body) were used. For imaging with the NIR camera, the mice were anaesthetised with isoflurane inhalation solution (Pfizer Inc., New York, USA). The imaging time points were 15–30 min, 1 h, 3 h, 5–6 h, and 1–18 days post-injection. After imaging at the time points of 3–4 h (n = 3), 3–4 days (n = 1), and 15–18 days (n = 4), the mice were anaesthetised and euthanised for dissection. In the dissection, blood was sampled from the vena cava by which the mice were put down. The NIR camera was used as a surgical guide for the removal of the BAT and beige-cell containing tissues. The excised adipose tissues and organs were fixed with either formalin or glutaraldehyde.

To confirm that the adipose tissue imaging with PMB-CNT did not depend on the sex of the mice, similar experiments were performed on male mice (BALB/cAJc1-nu/nu, Male; CLEA Japan, Inc.). The imaging time points were 0–10 min, 1 h, and 3 h (n = 3). After imaging at the 3 h post PMB-CNT-injection time, the mice were euthanised and dissected, and adipose tissues were removed and fixed with formalin.

These animal experiments were performed in accordance with the regulations and approval of the Animal Care and Use Committee of the institutes of National Institute of Advanced Industrial Science for female mice experiments and Technology and Hokkaido University for male mice experiments).

### Histological observations

Tissues fixed with formalin were sliced and stained with NFR and haematoxylin eosin (HE) solution. To identify BA and beige adipose cells, UCP-1 immunostaining was performed after activating the antigen with EDTA treatments (1 mM, pH 8.0, Kanto Chemical Co., Inc., Tokyo, Japan, Catalogue No. 14097-00) and heating at 95 °C for 20 minutes. Primary and secondary antibody reactions were performed using a rabbit anti-UCP-1 polyclonal antibody (Abcam Plc., Cambridge, UK, Catalogue No. ab10983) and Histstar^TM^ (Rb) for mouse tissue (Medical & Biological Laboratories Co., Ltd., Nagoya, Japan, Catalogue No. 8470). The observations were made with microscopy (Olympus BX60, Olympus Corp.) and NIR fluorescence microscopy.

To determine endothelial cells, CD31 immunostaining was performed using a rabbit anti-CD31 polyclonal antibody (Abcam Plc., Catalogue No. ab124432) and an Alexa Fluor^®^ 488 goat anti-rabbit IgG second antibody (Thermo Fisher Scientific Inc., Waltham, MA, USA, Catalogue No. A11008), along with Can Get Signal immunostain Solution B (TOYOBO Co., Ltd., Osaka, Japan, Catalogue No. NKB-601). The stained tissues were observed with confocal microscopy (LSM 5 PASCAL, Carl Zeiss AG, Oberkochem, Germany) at an excitation wavelength of 488 nm and NIR fluorescence microscopy.

### PMB-CNT/murine sera reactions

PMB-CNT (CNT: 0.5 mg/mL, 125 mL) was mixed with 50% diluted murine sera (125 mL) in a 1.5 mL tube, which was kept rotated at 4 °C overnight. Then, 1 mL of the reaction mixture was dropped onto a Matsunami Adhesive Silane (MAS)-coated glass slide (Matsunami Glass Ind., Ltd., Osaka, Japan) to prepare smeared samples. Immunostaining studies were then performed after fixation with 4% paraformaldehyde solution. Primary/secondary antibody reactions were carried out using a rabbit polyclonal anti-apolipoprotein B antibody (Abcam Plc., Catalogue No. ab20737), or a rabbit polyclonal anti-apolipoprotein E antibody (Abcam Plc., Catalogue No. ab83115) with Alexa Fluor^®^ 488 goat anti-rabbit IgG second antibody (Thermo Fisher Scientific Inc., Catalogue No. A11008), and a goat polyclonal anti-ApoC-II antibody (Q-20; Santa Cruz Biotechnology Inc., Santa Cruz, CA, USA, Catalogue No. sc-19014) with Alexa Fluor^®^ 488 chicken anti-goat IgG second antibody (Thermo Fisher Scientific Inc., Catalogue No. A21467). Normal rabbit IgG (Jackson ImmunoResearch Inc., West Grove, PA, USA, Catalogue No. 011-000-003) and normal goat IgG (Santa Cruz Biotechnology Inc., Catalogue No. sc02028) were used in the respective primary antibody reactions as controls. Additionally, the reaction supernatants were subjected to Western blotting studies using a rabbit polyclonal anti-apolipoprotein B antibody (Abcam Plc., Catalogue No. ab20737), a mouse monoclonal anti-apolipoprotein E antibody (Abcam Plc., Catalogue No. ab1906), or a rabbit monoclonal anti-bovine serum albumin antibody (Abcam Plc., Catalogue No. ab192603), along with a horseradish peroxidase-conjugated anti-rabbit or anti-mouse IgG secondary antibody (Cell Signaling Technology, Inc., Beverly, MA, USA) (21). In some experiments, Can Get Signal^®^ Immunoreaction Enhancer Solution (TOYOBO Co., Ltd., Catalogue No. NKB-101) was used. Chemiluminescence reactions were performed using ECL Western blotting detection reagents (GE Healthcare UK, Ltd., Buckinghamshire, England). Visualisation and intensity measurements of protein bands were performed using a C-DiGit^®^ chemiluminescent Western blot scanner (LI-COR, Inc., Lincoln, NE, USA).

### Transmission electron microscopy observation

For TEM observation, the tissues blocks were fixed with glutaraldehyde for about 3 h at 4 °C, washed with phosphate buffer (pH 7.4), and stained with an aqueous solution containing 1% OsO4 at 4 °C for 2 h. After washing with phosphate buffer (pH 7.4), the specimens were dehydrated using aqueous solutions of ethanol from 50% to 95%, finishing with 100% ethanol. The ethanol was then replaced, initially by n-butyl glycidyl ether and, secondly, by a mixture of n-butyl glycidyl ether and Epon812. Finally, the tissues were embedded in epoxy resin (Epon812) and sliced for the TEM observations. These observations were performed with an acceleration voltage of 80 kV (H7600 TEM, Hitachi, Ltd, Tokyo, Japan).

## Additional Information

**How to cite this article:** Yudasaka, M. *et al*. Near-Infrared Photoluminescent Carbon Nanotubes for Imaging of Brown Fat. *Sci. Rep.* **7**, 44760; doi: 10.1038/srep44760 (2017).

**Publisher's note:** Springer Nature remains neutral with regard to jurisdictional claims in published maps and institutional affiliations.

## Supplementary Material

Supplementary Information
