# Supplementary material for: Near-Infrared Photoluminescent Carbon Nanotubes for Imaging of Brown Fat
Source: Sci Rep. 2017 Mar 20;7:44760. doi: 10.1038/srep44760 (PMC5357894; doi:10.1038/srep44760)
Supplement: Supplementary Information [file srep44760-s1.pdf]

# Supplementary Information

## Near-Infrared Photoluminescent Carbon Nanotubes for Imaging of Brown Fat

Masako Yudasaka[1], Yohei Yomogida[1], Minfang Zhang[2], Takeshi Tanaka[1], Masako Nakahara[3], Norihiko Kobayashi[3], Yuko Okamatsu-Ogura[4], Ken Machida[4], Kazuhiko Ishihara[5], Kumiko Saeki[3], Hiromichi Kataura[1]

[1]Nanomaterials Research Institute, National Institute of Advanced Industrial Science and Technology, Tsukuba, Ibaraki 305-8565, Japan
[2]CNT-Application Research Center, National Institute of Advanced Industrial Science and Technology, Tsukuba, Ibaraki 305-8565, Japan
[3]Department of Disease Control, Research Institute, National Center for Global Health and Medicine, Shinjuku-ku, Tokyo 162-8655, Japan
[4]Department of Biomedical Sciences, Graduate School of Veterinary Medicine, Hokkaido University, Sapporo 060-0818, Japan
[5]Department of Materials Engineering, The University of Tokyo, Hongo, Tokyo 113-8656, Japan

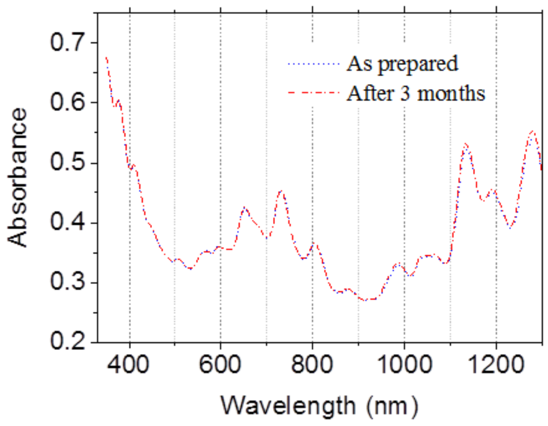


**Supplementary Figure 1.** Absorption spectra of PMB-CNT aqueous dispersion solutions as-prepared (blue line) and after being refrigerated for 3 months (red line). Concentrations of CNT were 0.05 mg/mL, which is close to the initial concentration of CNTs in mouse blood following the

intravenous injection.

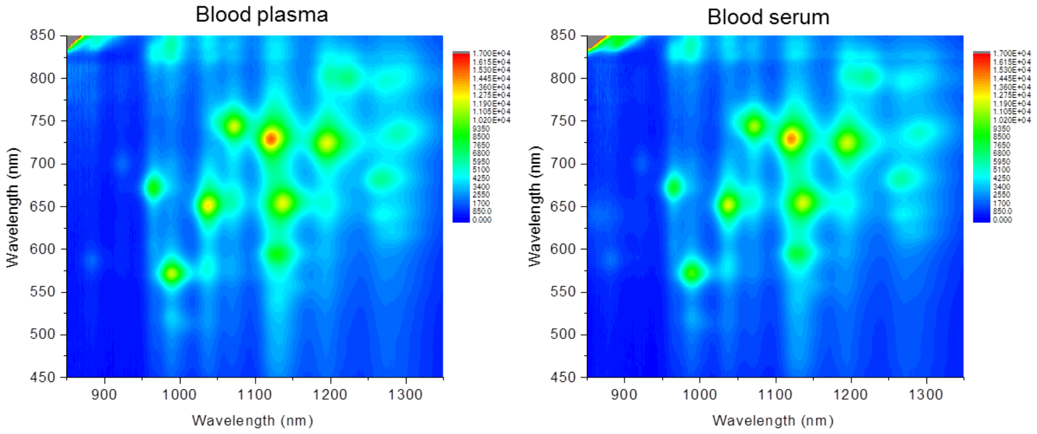


**Supplementary Figure 2.** Photoluminescence spectra of aqueous dispersion solutions of PMB-CNT (0.2 mL) mixed with mouse blood serum (2 mL) and plasma (2 mL) after being left at room temperature for 24 h.

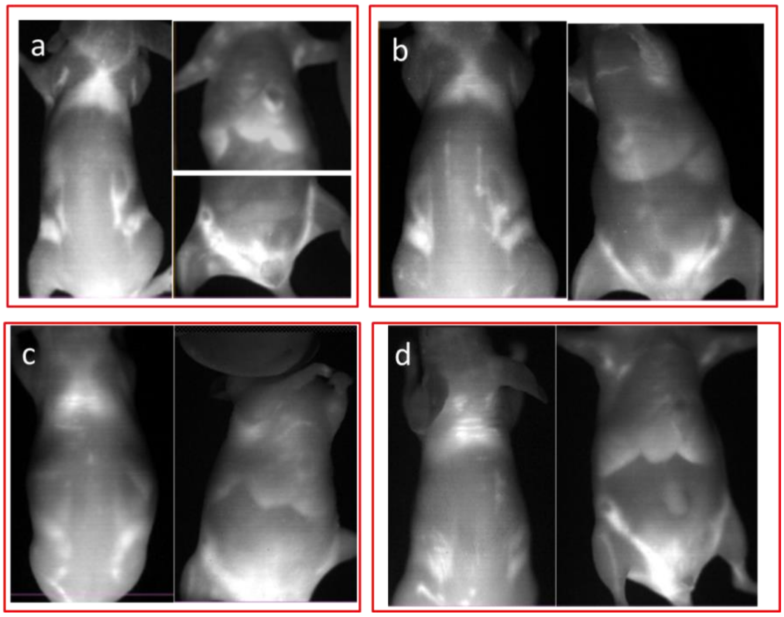


**Supplementary Figure 3.** NIR photoluminescence (PL) whole body images of four female mice into which PMB-CNT was intravenously injected via tail veins. The post-injection times were 5 h (a) and 3 h (b-d). Bright signals appeared in the liver interscapular, paravertebral, axillar, retroperitoneal, inguinal, and pelvic areas (n = 8). The latter six areas are known to contain

adipose tissues. Bright areas within the bowels are due to fluorescent ingredients within the food.

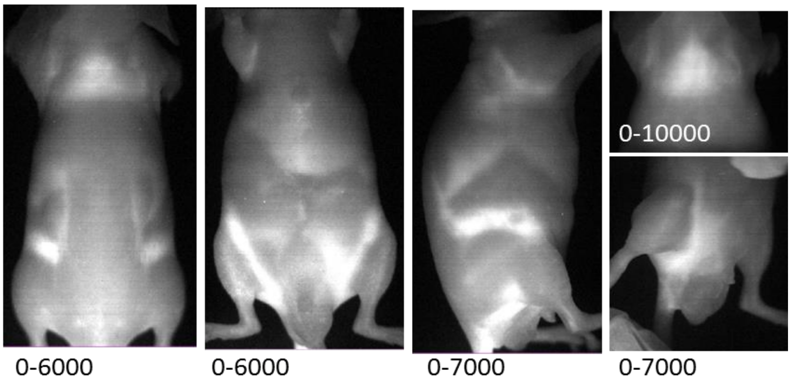


**Supplementary Figure 4.** NIR PL whole body images of PMB-CNT-injected male mice at 3 h post-injection. Regions of adipose tissue and liver appeared bright (n = 3), similar to those in female mice (Figure 1 and Supplementary Figure 3).

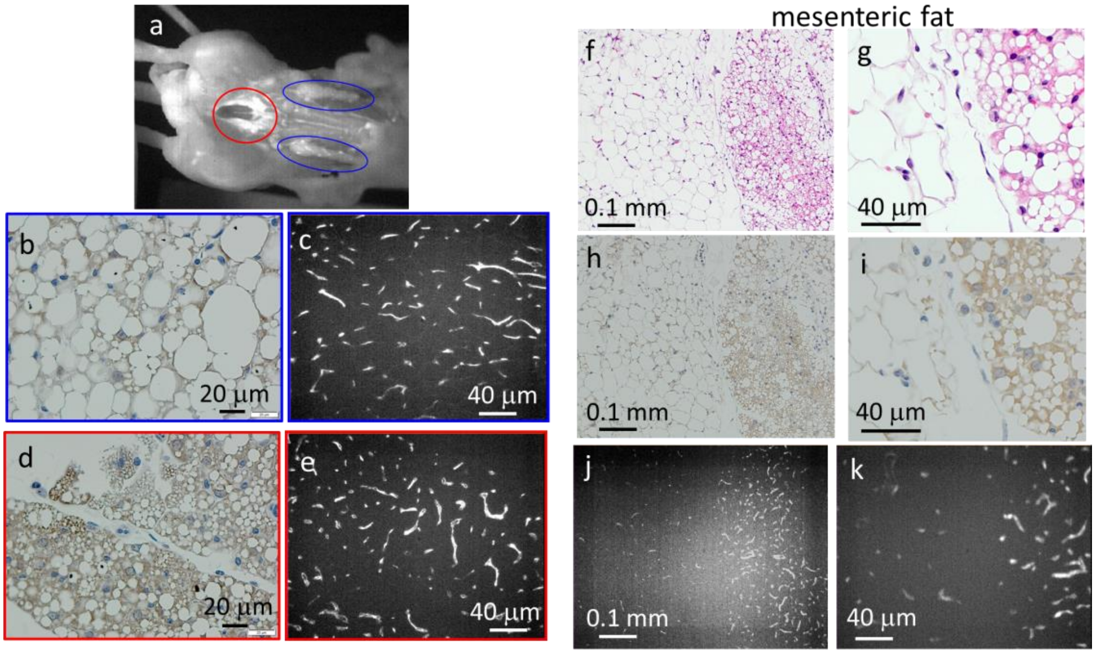


**Supplementary Figure 5**. Histological observation of retroperitoneal, gonadal, and mesenteric fat depots that appeared bright under NIR PL whole body imaging. NIR image of the gastrointestinal tract removed from a

female mouse at 3.5 h post-intravenous injection of PMB-CNT (a). Blue and red ellipses in (a) denote retroperitoneal (b, c) and gonadal (d, e) fat depots, respectively. Mesenteric fat depots are shown in f-k. Tissues were immunostained using anti-UCP-1 antibody along with Mayer's haematoxylin solution counterstaining (b, d, h, i). The other staining techniques were HE (f, g), nuclear fast red (c, e), and no staining (j, k). Visible light micrographs (b, d, f-i) and NIR PL micrographs (c, e, j, k). In the mesenteric fat depots (f, g), areas with both high and low accumulations of CNTs were observed (j, k). The high accumulations was in beige-like morphology (g), as evidenced by anti-UCP1 antibody staining (h, i).

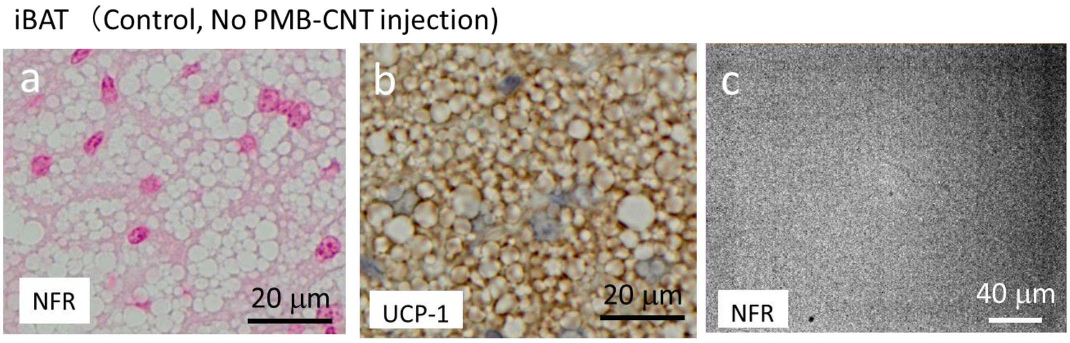


**Supplementary Figure 6**. Optical micrographs of interscapulum BAT (iBAT) from a control mouse (Female n = 3) stained with NFR (a, c) and UCP-1 (b). Visible light micrographs (a, b) show the characteristic structure of iBAT. NIR PL micrograph (c) does not show any bright spots, indicating that no substance that emits NIR PL is present in the iBAT of control mice.

A

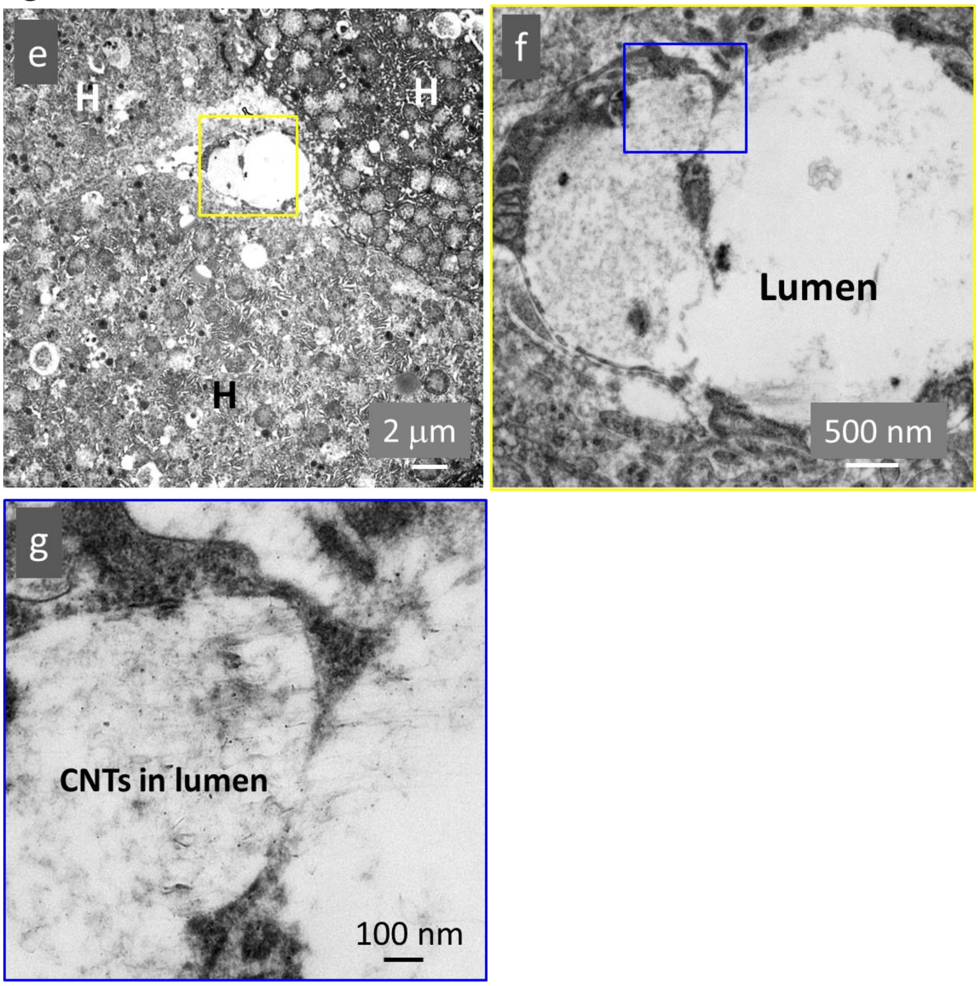

B

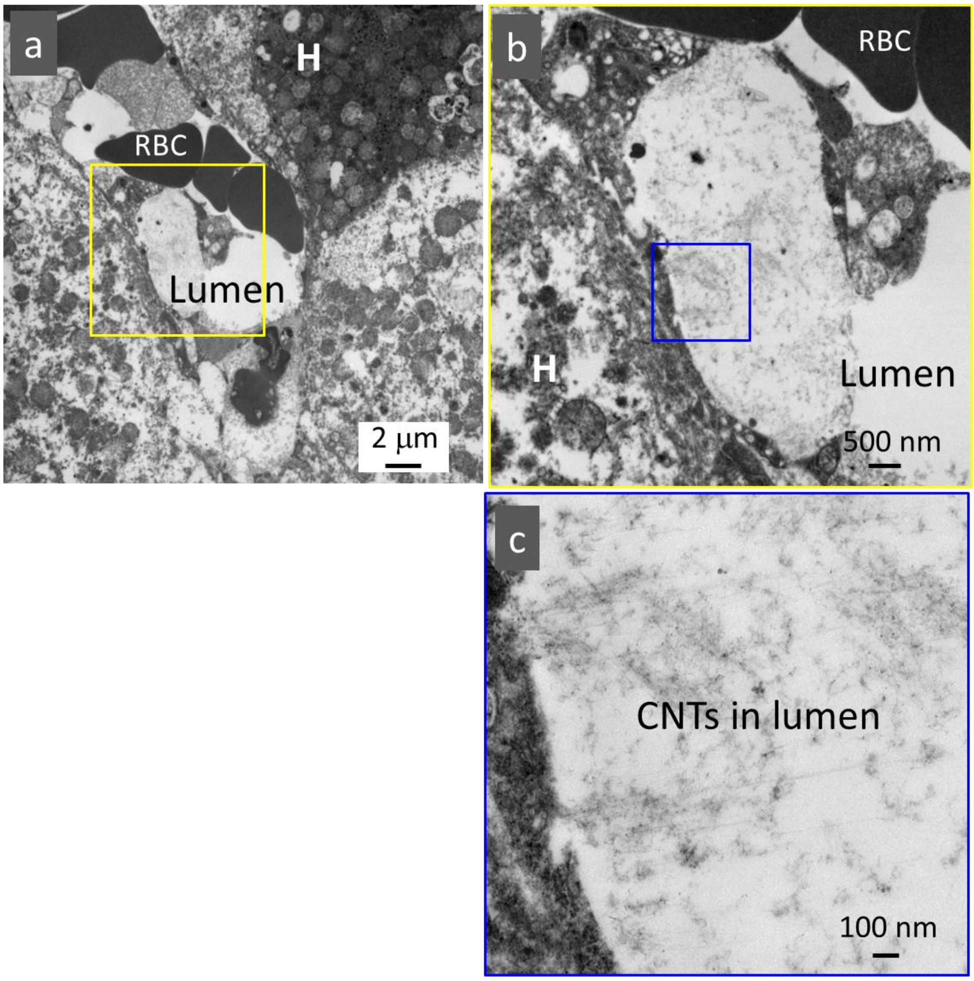
a
H
RBC
Lumen
2 µm
b
RBC
H
Lumen
500 nm
c
CNTs in lumen
100 nm

C

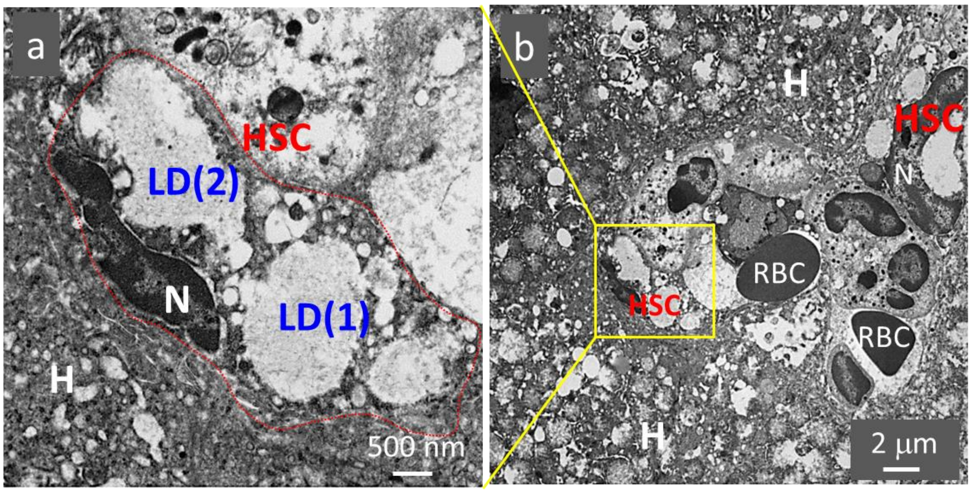
a
HSC
LD(2)
N
LD(1)
H
500 nm
b
H
HSC
N
RBC
HSC
RBC
H
2 µm

**D**

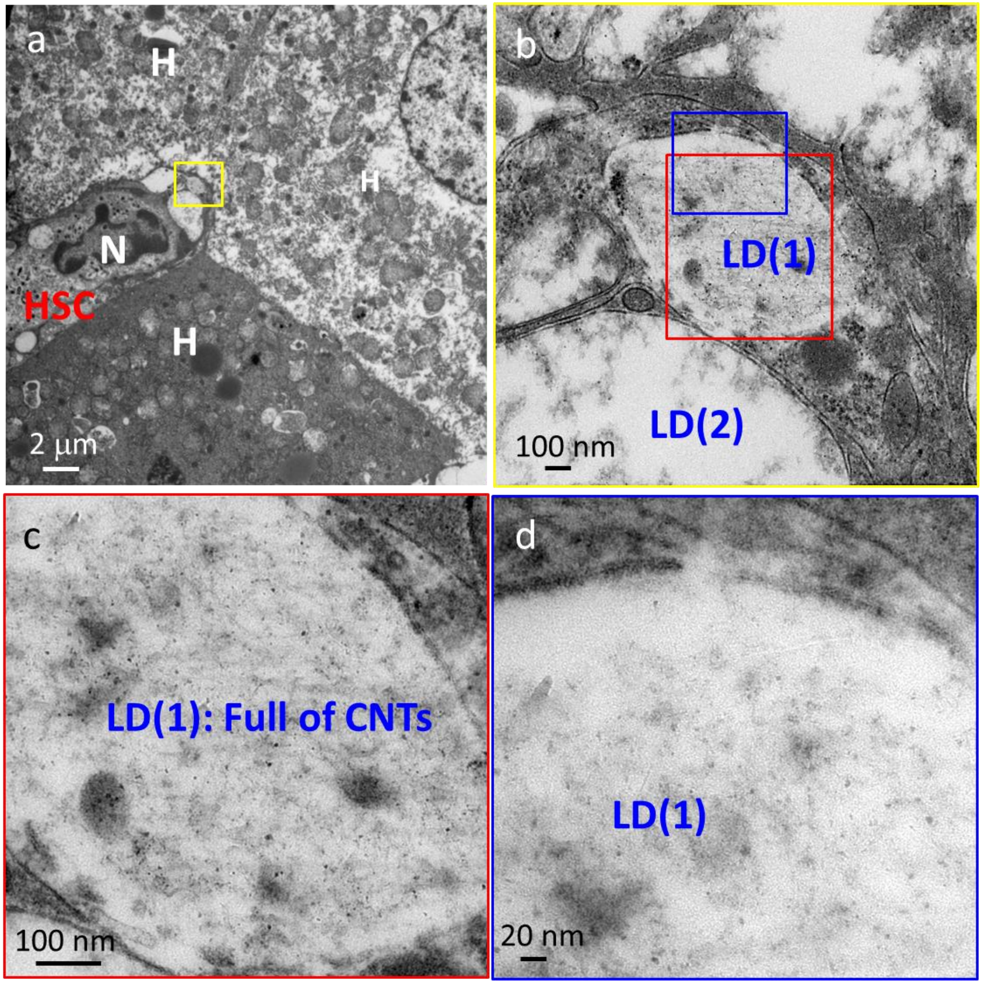


**Supplementary Figure 7.** TEM images of liver at 3.5 h post-PMB-CNT injection. CNTs are visible as dark fibre-like objects in the HSC lipid droplets (**A**, **B**) and sinusoidal lumens (**C**, **D**). HSC: Hepatic stellate cell. H: Hepatocyte. N: Nucleus. LD(1), LD(2): Lipid droplets.

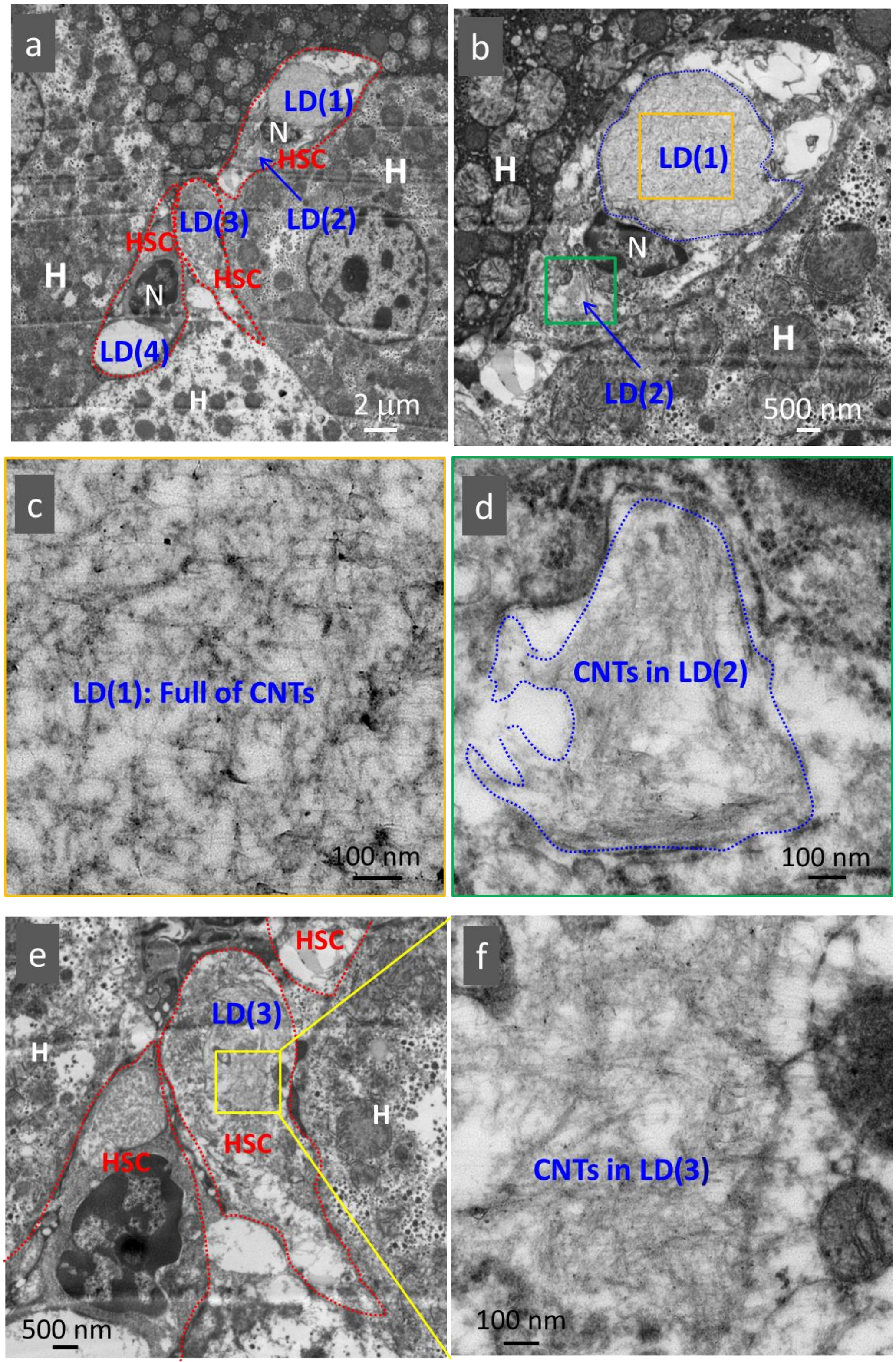


**Supplementary Figure 8.** TEM images of liver tissue at 14 days post-PMB-CNT injection. Hepatic stellate cells (HSCs) are localized. CNTs are visible as dark fibre-like objects in the HSC lipid droplets, LD(1), LD(2), and LD(3).

CNTs were not localized in the sinusoidal lumen. Red line: HSC, blue line: area containing CNTs.

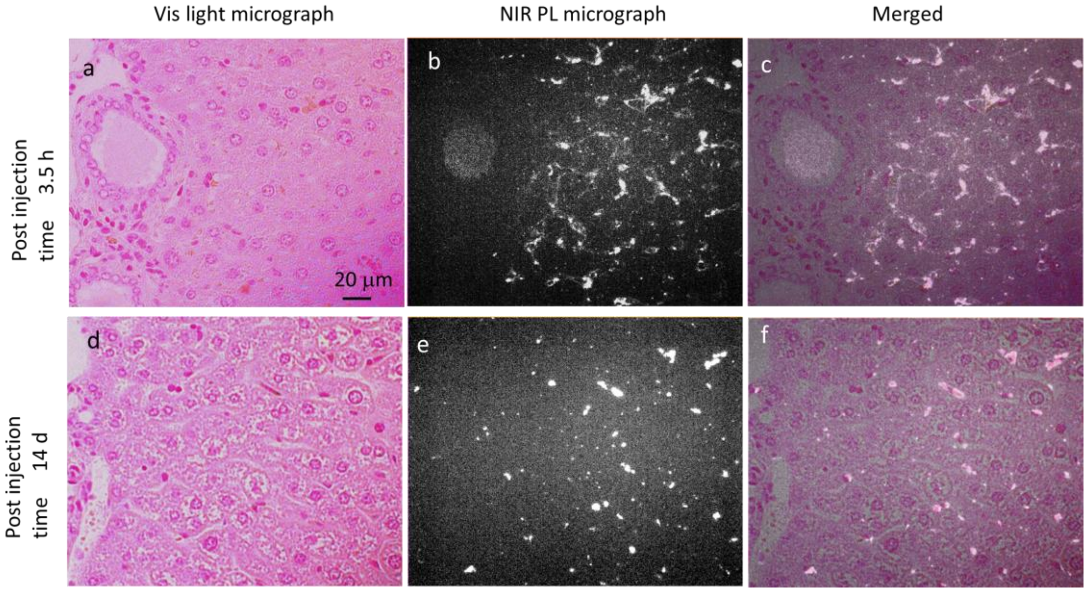


**Supplementary Figure 9.** Visible light and NIR PL micrographs of liver tissue at 3.5 h (a-c) and 14 d (d-f) post-PMB-CNT injection. Tissues were stained with nuclear fast red. Referring to the TEM observation (Supplementary Figures 7, 8) and the fact that the CNT quantity in the liver did not change from 3.5 h to 14 d post-injection, it is estimated that the CNT found in the sinusoidal lumen at 3.5 h moved to the lipid droplets of HSC. The CNT quantities were estimated from optical absorbance of liver tissue lysates at 700 nm.

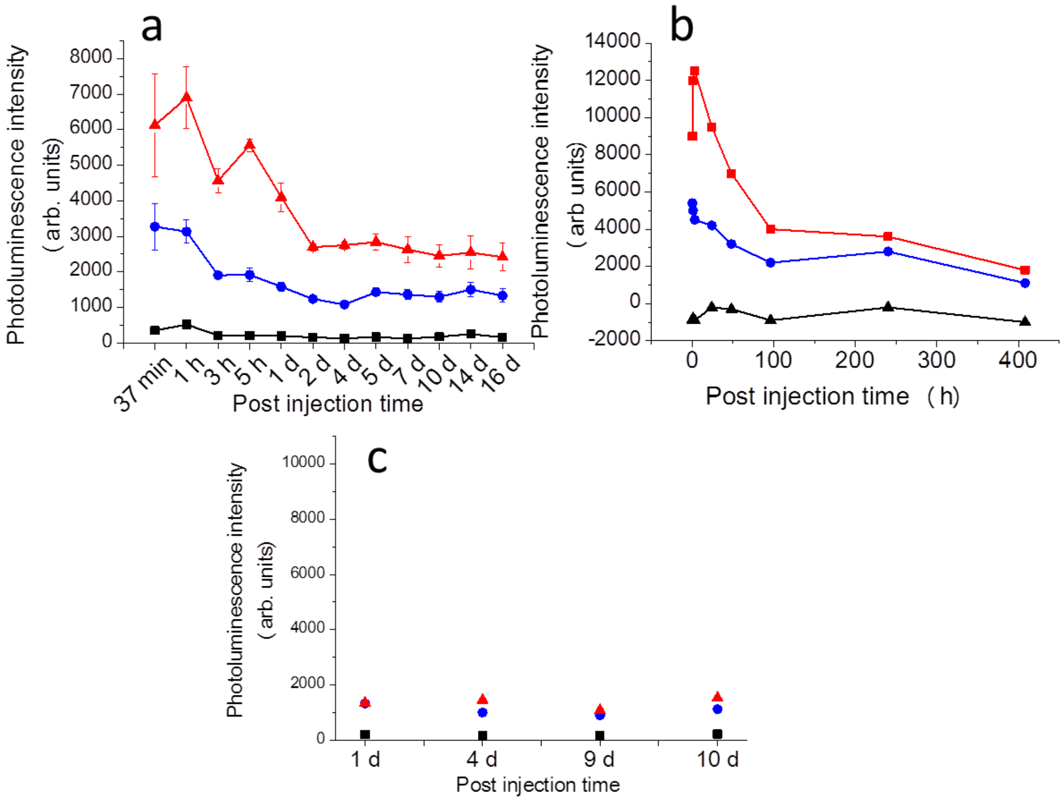


**Supplementary Figure 10.** Time courses of photoluminescence (PL) intensity. Mice with (a, b) or without (c) PMB-CNT injection via tail veins were subjected to NIR PL imaging. PL intensities were measured in iBAT (red lines) and non-iBAT (blue lines) areas of the scapulae. Background PL intensities (black lines) were measured on the plate on which mouse was put. The NIR PL intensity of PMB-CNT in iBAT increased in the initial phase (< 1 h) and then decreased with time. Control mice without PMB-CNT injections showed only low PL intensity.

A (iBAT, Day 14)

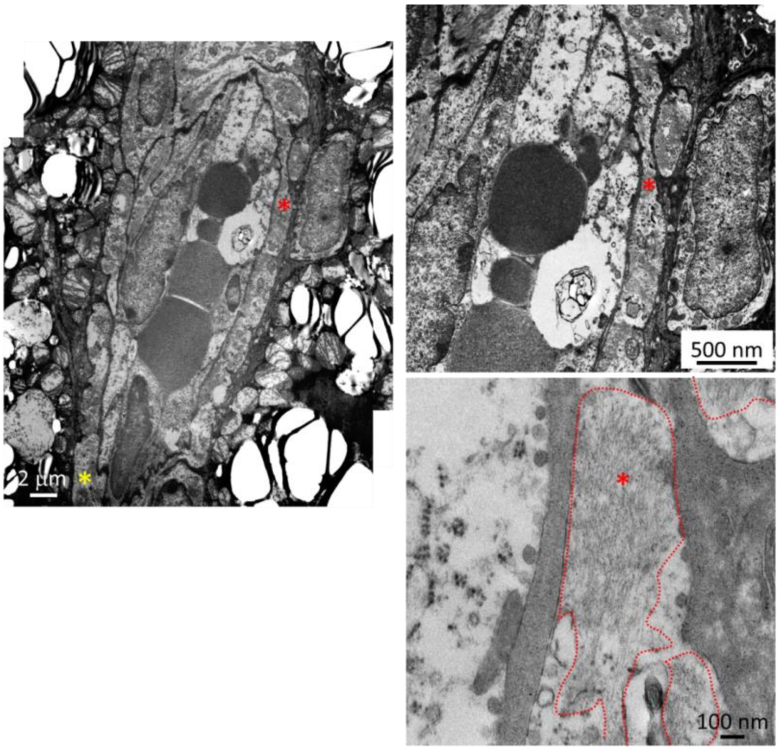


B (iWAT, Day 14)

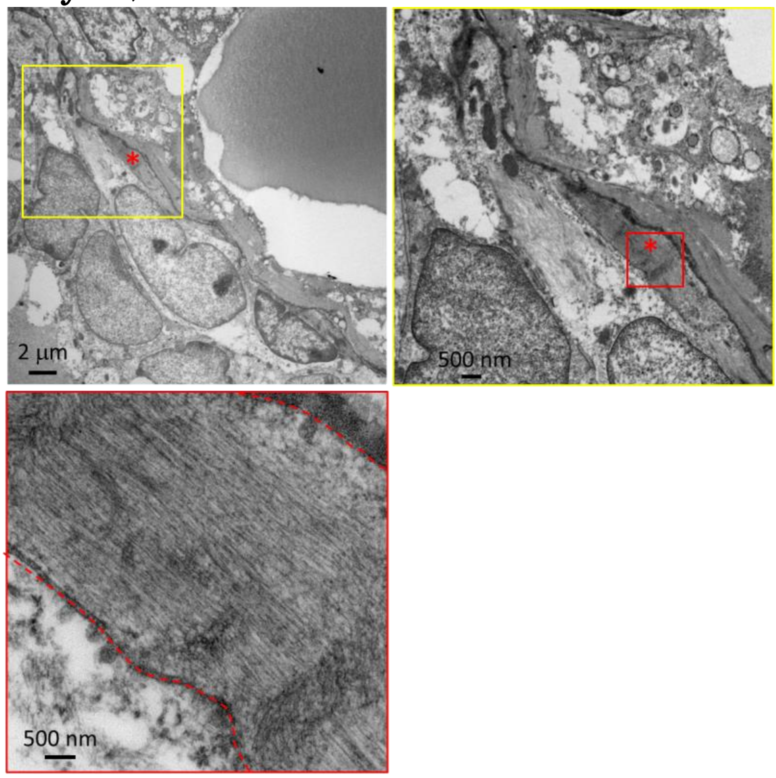


**Supplementary Figure 11.** TEM images of iBAT **(A)** and iWAT **(B)** at 14 days post-injection. The broken red lines denote sites where densely packed CNTs

were localized. CNTs were commonly detected in thick bundles at 14 days post-injection.
